# Genome-Wide Discovery of Structural Variants Reveals Distinct Variant Dynamics for Two Closely Related *Monilinia* Species

**DOI:** 10.1093/gbe/evad085

**Published:** 2023-05-22

**Authors:** Muhammed R Durak, Hilal Ozkilinc

**Affiliations:** School of Graduate Studies, MSc Program in Molecular Biology and Genetics, Çanakkale Onsekiz Mart University, Çanakkale, Turkey; School of Graduate Studies, MSc Program in Molecular Biology and Genetics, Çanakkale Onsekiz Mart University, Çanakkale, Turkey; Department of Molecular Biology and Genetics, Faculty of Sciences, Çanakkale Onsekiz Mart University, Çanakkale, Turkey

**Keywords:** structural variants, copy number variation, comparative genomics, *Monilinia* spp

## Abstract

Structural variants (SVs) are variants with sizes bigger than 50 bp and capable of changing the size, copy number, location, orientation, and sequence content of genomic DNA. Although these variants have been proven to be extensive and involved in many evolutionary processes along the tree of life, there is still insufficient information on many fungal plant pathogens. In this study, the extent of SVs, as well as single-nucleotide polymorphisms (SNPs), has been determined for two prominent species of the *Monilinia* genus (the causal agents of brown rot disease in pome and stone fruits): *Monilinia fructicola* and *Monilinia laxa* for the first time. The genomes of *M. fructicola* were found to be more variant-rich in contrast to *M. laxa* based on the reference-based variant calling (with a total number of 266.618 and 190.599 SNPs and 1,540 and 918 SVs, respectively). The extent, as well as distribution of SVs, presented high conservation within the species and high diversity between the species. Investigation of potential functional effects of characterized variants revealed high potential relevance of SVs. Moreover, the detailed characterization of copy number variations (CNVs) for each isolate revealed that around 0.67% of *M. fructicola* genomes and 2.06% of *M. laxa* genomes are copy number variables. The variant catalog as well as distinct variant dynamics within and between the species presented in this study opens doors for many further research questions.

**Significance**

Structural genomic variants have important effects on both evolutionary biology and functional consequences for important adaptive traits. Information on structural variants (SVs) of fungal plant pathogens is scarce, especially within the species. This study documented for the first time the extent, distribution, and potential functional consequences of SVs for within and between of two important plant pathogenic species, *Monilinia fructicola* and *Monilinia laxa*. Overall results suggested potential differing evolutionary processes for these two closely related species. While this study sheds light on the structural genomic features of these agriculturally devastating pathogens, it also opens up avenues for further research to understand the evolutionary implications of these findings in disease management.

## Introduction

Characterizing the content and effects of genomic variations along the tree of life is a long-lasting task in evolutionary genetics. Single-nucleotide polymorphisms (SNPs) and structural variations (SVs) are the main sources of genomic variability, and SNPs were thought to be the predominant form of variation for a long time (Freeman, et al. 2006; Sachidanandam, et al. 2001). On the contrary, genomic analyses in organisms including humans, *Drosophila*, and mice have shown that SVs comprise a larger proportion of the total genetic variation compared with SNPs (e.g., Auton, et al. 2015; Zichner, et al. 2013; Yalcin, et al. 2011). Also, many recent studies have shown that structural variations are not only extensively abundant and also associated with many differences in phenotypes (Divashuk, et al. 2019; Wang, et al. 2020; Zhao and Gibbons 2018). Thus, the discovery of genomic variants is one of the key steps in understanding the many possible evolutionary outcomes and phenotypic differences for populations. Although the definition differs in different studies, SVs are mainly defined as variants that change the size, copy number, location, orientation, and sequence content with a size >50 bp (Escaramís, et al. 2015). The recognized class of SVs comes in many different shapes and forms and includes many different types of genomic events, including deletions, duplications, novel insertions, inversions, mobile element transpositions, and translocations (Periwal and Scaria 2015). These variants, in general, are subdivided into two groups based on their impact on the sequence content: balanced, where no gain or loss of genetic material occurs (i.e., inversions and translocations), and unbalanced, where part of genome is lost/deleted or duplicated (i.e., copy number variation, CNV) (Collins, et al. 2017). Interpretation of SVs has been difficult in the past due to limitations on the sequencing technologies, bioinformatics approaches, and their functional consequences (Fan, et al. 2014; Weischenfeldt, et al. 2013). The number of tools to detect SVs has been increasing in recent years based on the demand for SV detection and the increase in the data to analyze (Lei, et al. 2022). In human genetics, SVs have been extensively characterized and many studies have shown the direct association of these variants with many chronic diseases (Feuk , Carson, et al. 2006; Yi and Ju 2018). There is also an increasing number of studies showing the content and prevalence of SVs concerning different phenotypes in many different organisms, including plants and animals (e.g., Hämälä, et al. 2021; Hou, et al. 2012; Kadri, et al. 2014). However, for fungal plant pathogens, little or no information is known about the full extent of SVs and how different types of SVs vary within and between different species. The known studies on this subject are generally focused on a certain type of SV (such as only CNV or TE) (e.g., Muszewska, et al. 2019; Zhao and Gibbons 2018). Besides, similar to other SV types, the number of studies that investigates the CNV in fungal pathogens is relatively much smaller than in other organisms and even the extent of these common SV type is not known for many fungal plant pathogen species. As an example, the contribution of CNV to genetic diversity in 71 isolates of *Aspergillus nidulans* has been characterized and comparative genomic approaches have been used to present the CNV profile (Zhao and Gibbons 2018). This study has shown that ∼10% of the genome is CN variable and these CNVs are nonrandomly distributed around the genes related to transposable elements and secondary metabolism functions (Zhao and Gibbons 2018).

These variants have also recently been shown to have a potential role in the adaptive evolutionary processes of fungal pathogenic species (Badet, et al. 2021; Langner, et al. 2021). For example, a study on wheat pathogens *Parastagonospora nodorum*, *Pyrenophora tritici-repentis*, and *Bipolaris sorokiniana* showed that effector gains through horizontal gene transfer which results in novel insertions into the genome caused specialization to new hosts (McDonald, et al. 2019). Another study conducted on well-studied wheat pathogen *Zymoseptoria tritici* has shown that transposable element insertions affect the genetic regulation of melanin production (Krishnan, et al. 2018). In another study conducted on powdery mildew pathogen, *Erysiphe necator*, the isolates collected from vineyards exposed to the azole fungicides showed CNV in the *CYP51* gene, the target region of azole fungicides (Jones, et al. 2014).

*Monilinia* spp. is a genus of *Ascomycete* fungi in the family Sclerotiniaceae and includes pathogenic species that cause brown rot disease on stone and pome fruits worldwide (Byrde and Willetts 2013; Holb 2008; Hrustić, et al. 2013; Ozkilinc, et al. 2020). For the most common and widely studied pathogenic species *Monilinia fructicola*, *Monilinia laxa*, and *Monilinia fructigena*, high-quality genome data for only one isolate of each of these three species are available in NCBI (De Miccolis Angelini, et al. 2019; Landi, et al. 2018; Naranjo-Ortíz, et al. 2018). Genome-wide studies for these species are mainly focused on questions concerning virulence, host–pathogen interactions, and fungicide resistance (Jones, et al. 2014; Marcet-Houben, et al. 2021; Vilanova, et al. 2021). However, neither the genome-wide characterization of structural variations nor their effects within the species level are known. In a recent study by De Miccolis Angelini et al. (2022), genome comparisons for *M. fructicola*, *M. laxa*, and *M. fructigena* have been presented using a single isolate from each species. However, genome data from larger numbers of isolates for each species are needed to explore and better understand the enormous diversity in fungal pathogen populations. In this study, we aimed to comprehensively characterize the extent of genome-wide SVs with their potential functional effects within and between two prominent species of the *Monilinia* genus: *M. fructicola* and *M. laxa*. This work enables SVs to be identified in many aspects (such as their sizes, localizations, and functional effects across genomes) by comparisons of intra- and interspecies levels for these important fungal pathogens. Furthermore, these findings open up research on their potential roles in adaptive evolutionary processes.

## Results

### Genome Assemblies

#### Reference-Based Genome Assembly Statistics

Reference-based genome assembly was performed on one isolate per species (Ti-B3-A3-2 for *M. fructicola* and Yildirim-1 for *M. laxa*) based on the well-assembled reference genomes available for *M. fructicola* (De Miccolis Angelini, et al. 2019) and *M. laxa* (Landi, et al. 2020) to discover the SNPs and SVs of the selected isolates by using bioinformatics pipelines that use mapping strategies. The assembly statistics of reference-based genome assembly were found to be the same as the references used with 20 and 49 scaffolds: 2.5 and 2.4 Mb N50 size, 7 and 8 L50, 0 N's per 100 kb, 40.81 and 39.81 GC content (%), and 44 and 42 Mb in size for *M. fructicola* and *M. laxa*, respectively.

#### De Novo Genome Assembly Statistics

De novo genome assemblies for each selected isolate were generated by using SPADES version 3.11.1 (Bankevich, et al. 2012) to investigate the synteny within the species. In general, genomes of *M. laxa* presented an average of 155 contigs and an N50 length of 552 kilobases (kb). On the other hand, the genomes of *M. fructicola* isolates presented an average of 679 contigs and 131 kb for N50 length. The total lengths of the assembled *M. laxa genomes* were similar to each other with sizes ranging from 41.99 to 42.16 Mb in length. The total length of the *M. fructicola* isolates ranged between 43.84 and 44.51 Mb. Genome assembly quality statistics of each isolate and the completeness of each de novo assembled genomes are presented in supplementary table S1, Supplementary Material online. In addition, coverage histograms for each de novo assembled genome are presented in supplementary figure S1, Supplementary Material online.

### Mapped Illumina Reads

The Illumina reads of each selected isolate were mapped to the reference genomes (Ti-B3-A3-2 for *M. fructicola* and Yildirim-1 for *M. laxa* isolates) obtained from reference-based genome assembly. Reads were mapped with percentages ranging from 83.70% to 92.17% for *M. fructicola* isolates, whereas these percentages were greater for *M. laxa* isolates, ranging from 96.69% to 99.01%. The average mean coverages were 118 and 154 for *M. fructicola* and *M. laxa*, respectively. Number of reads, overall properly paired read percentages, and mean coverages with average values for each alignments are presented in supplementary table S2, Supplementary Material online. These high average coverages allowed the precise identification of variants for each isolate.

### The Abundance, Distribution, and Prediction of Functional Effects of SNPs and SVs Along the Genomes of *M. fructicola* and *M. laxa*

**Fig. 1.**
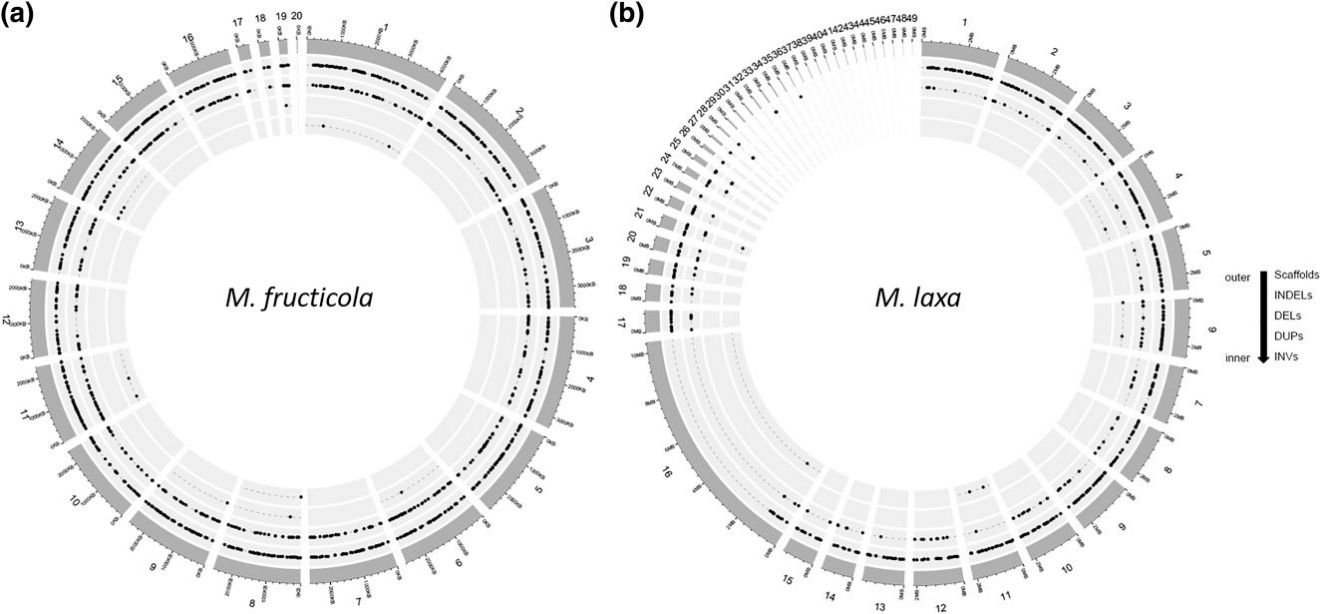
Distribution of SVs along the genomes of *M. fructicola* and *M. laxa*. The circular plots showing the distribution of discovered structural variations (INDELs, deletions [DELs], duplications [DUPs], and inversions [INVs] from outer to inner circle as named in the legend) based on variant calling from all isolates along the genome of (*a*) *M. fructicola* and (*b*) *M. laxa*. INDELs (second inner circle) were the most common SV type, and the number of duplications (third inner circle) and inversions (forth inner circle) was extremely low for both species. At least one type of SV was present at each scaffold of *M. fructicola* genomic data set. Scaffolds between 37 and 49 and a large proportion of scaffold 16 for *M. laxa* did not contain any type of SVs.

The distribution and the density of the variants were highly variable among the scaffolds and the species. Especially, representations of SVs along the genomes were highly variable between the species. The circular plots showing the abundance and distribution of discovered SVs along the genomes are presented in figure 1*a* and *b*. Compared with *M. fructicola*, *M. laxa* presented a highly variable distribution of the variants across its genomic regions, with some regions showing conservation (fig. 1*a* and *b*).

Genome sizes of isolates on average were highly similar within each species (44 Mb for *M. fructicola* and 42 Mb for *M. laxa*). However, the number of both SNPs and SVs was around 1.5 times higher for *M. fructicola* genomic data set compared with that for *M. laxa* (table 1). Few duplication and inversion events were identified for both species (table 1); however, this result needs to be taken cautiously due to the strict filtering parameters that were applied to eliminate the false-positive variants.

**Table 1:** Total Number of SNPs and SVs, Number of SVs by Type (INDELs, Deletions, Duplications, and Inversions), and the Variant Ratio between Species

Type of Variant	*M. fructicola*	*M. laxa*	Variant Ratio (MF/ML)^a^
	Number of Variants		
SNPs	266.618	190.599	1.40
Total SV	1.540	918	1.67
INDELs	964	740	
Deletions	564	171	
Duplications	7	3	
Inversions	5	4	

a Variant ratio, number of variants in *M. fructicola*/number of variants in *M. laxa*.

The transition (Ts)/transversion (Tv) ratio in *M. laxa* was almost 2.3 times greater than in *M. fructicola* (the ratios were 5.93 and 2.53 for *M. laxa* and *M. fructicola*, respectively). As expected, the substitution rates of transitions were found to be higher than transversions for both species.

**Fig. 2.**
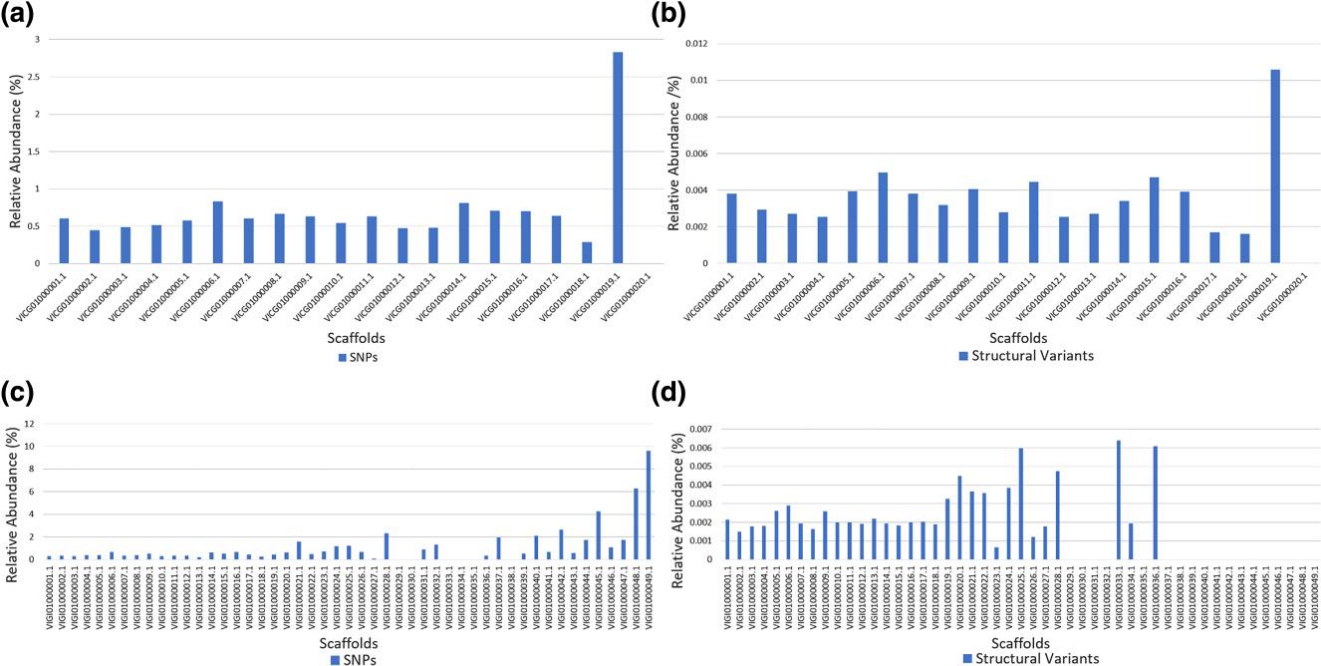
Abundance distribution of variants for each scaffold of *M. fructicola* and *M. laxa*. The relative abundances of (*a*) SNPs and (*b*) SVs along the 20 scaffolds for *M. fructicola* and (*c*) SNPs and (*d*) SVs along the 49 scaffolds for *M. laxa* genomic data sets.

The variant distributions among the scaffolds for *M. fructicola* were represented as bar graphs in figure 2*a* and *b*. The relative abundance of SNPs was the lowest at scaffold 18 (2.8%) and 20 (0.03%) (with scaffold names VICG010000018/20.1) for *M. fructicola* genomic data set (supplementary table S3, Supplementary Material online). The relative abundance of both SNPs and SVs was the highest at scaffold 19 (with scaffold name VICG010000019.1) with 7,219 SNPs and 27 SVs in it (supplementary table S3, Supplementary Material online). Interestingly, there was only a single SNP at scaffold 20 (VICG010000020.1) and no SV was present at this scaffold (supplementary table S3, Supplementary Material online). The single SNP at scaffold 20 was only present in the isolate coded BG-B1-A8, and the other isolates did not have any SNPs in this scaffold.

The distribution of SNP and SV variants differed among the scaffolds of *M. laxa* (fig. 2*c* and *d*). Scaffold 33 (with a size of 62.38 kb) did not contain any SNPs in it (supplementary table S4, Supplementary Material online). Moreover, many other scaffolds numbered 29, 30, 34, 35, and 38 with sizes varying between 70 and 24 kb had a low number of SNPs (supplementary table S4, Supplementary Material online). The relative abundance of SNPs was the highest at scaffold 49 among all, and this scaffold did not contain any SV (supplementary table S4, Supplementary Material online). In general, the distribution of SVs among the scaffold was variable for *M. laxa* genomic data set. For example, a total of eighteen scaffolds with sizes varying between 70 and 5 kb did not contain any SV in them. Moreover, three scaffolds (named 30, 35, and 38 with 69, 49, and 24 kb length, respectively) had only one SNP in them. Further investigations revealed that these scaffolds containing a unique SNP were found only in certain different isolates among the eight isolates studied. For example, a unique SNP seen in scaffold 30 was only detected in the Ni-B3-A2 isolate of *M. laxa*.

The prediction of functional effects of variants for the *M. fructicola* genomic data set showed that intergenic regions contained most of the SNPs (47.36% of all SNPs) (table 2). The second most common location of SNPs for *M. fructicola* was the downstream and upstream regions of the genes with 24.02% and 23.05%, respectively (Table 2). Similarly, most of the SNPs (41.02%) were located in intergenic regions throughout the *M. laxa* genome, with 27.87% of these SNPs located downstream and 25.59% upstream of genes (table 2). Since most SNPs were not located within the genic regions, a small proportion of SNPs with potential functional impact (e.g., lost/gain of a start codon) were identified (table 2).

**Table 2:** The Count and Percent of SNPs Based on the Prediction of Functional Effects by Main Types in Alphabetical Order for *M. Fructicola* and *M. Laxa*

Annotation	*M. fructicola*		*M. laxa*	
	Count	Percent (%)	Count	Percent (%)
Downstream gene variant	122.811	24.02	115.828	27.87
Intergenic region	242.133	47.36	170.458	41.02
Intron variant	5.785	1.14	4.332	1.04
Missense variant	11.685	2.28	9.653	2.32
Splice region variant	922	0.18	630	0.15
Synonymous variant	8.941	1.74	7.430	1.78
Upstream gene variant	117.874	23.05	106.345	25.59

In addition, the prediction of functional effects of SVs for *M. fructicola* showed that 57.79% of the SVs were found in the regions with transcript sequences (table 3). Only 0.31% of the total SVs were intronic variants (table 3). SVs were also found to be in both the upstream and downstream regions of the genes (table 3). Similarly, in the *M. laxa* genomic data set, 61.48% of total SVs were found to correspond to transcript sequences and only 0.31% of total characterized SVs were intronic variants (table 3).

**Table 3:** The Count and Percent of SVs Based on the Prediction of Functional Effects by Main Types in Alphabetical Order for *M. fructicola* and *M. laxa*

Annotation	*M. fructicola*		*M. laxa*	
	Count	Percent (%)	Count	Percent (%)
Downstream gene variant	772	5.85	1.195	5.34
Intergenic region	1.444	10.95	885	3.96
Intron variant	41	0.31	70	0.31
Transcript ablation	7.615	57.79	13.742	61.48
Upstream gene variant	786	5.96	1.306	5.84

### CNV in the Genomes of *M. fructicola* and *M. laxa*

**Fig. 3.**
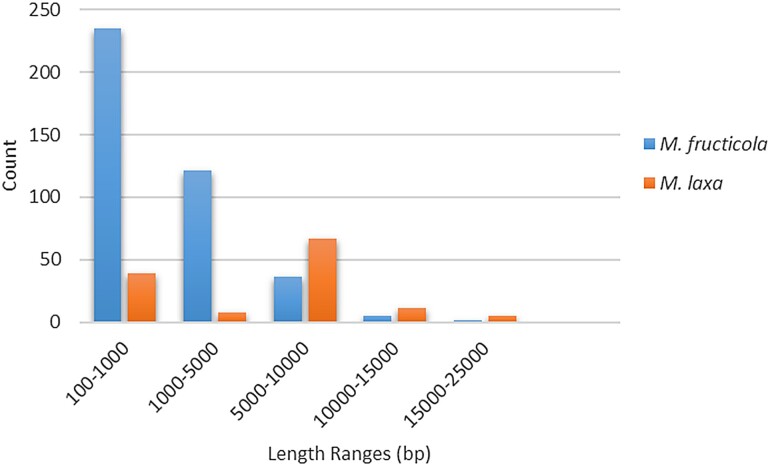
CNV sizes for *M. fructicola* and *M. laxa*. The size distribution of copy number variable regions (deletions and duplications) for (*a*) *M. fructicola* and (*b*) *M. laxa* isolates.

In addition to the total SVs, the CNVs were retrieved as deletions and duplications from the SVs for each isolate. The number of CNVs varied among isolates within species, with 136–210 in *M. fructicola* and 60–88 in *M. laxa* (table 4). In addition, considering the percent areas of ​​CNVs in table 4, the sizes covered by CNVs in the genomes also differed among the isolates. Overall, 0.67% of genomes for *M. fructicola* and 2.6% for *M. laxa* were found to have the effect of CNV variations (table 4). Evaluating the sequence lengths (in base pairs) affected by CNV across the genomes showed that CNVs of *M. laxa* isolates contained larger deletions/duplications in size compared with those from *M. fructicola* (fig. 3).

**Table 4:** The Number of Copy Number Variable Regions, Base Pairs Affected, and the Percent (%) of Copy Number Variable Regions for Each Isolate

Species	Isolate Code	Number of CNVs	Base pairs affected (bp)	Genome Size (Mb)	% of the Genome
*M. fructicola*	B5-A4	160	354.560	43.91	0.80
	BG-B1-A8	136	237.546	44.51	0.53
	BG-B3-A1	162	276.370	43.84	0.63
	BO-B3-A1	132	229.826	44.18	0.52
	SC-B2-A3	142	273.663	44.18	0.61
	SC-B2-A4	164	332.562	44.20	0.75
	TB1-A5	178	329.094	44.23	0.74
	YK-1	210	373.390	43.88	0.85
	**Average**	**160.5**	**300**.**876**	**44**.**11**	**0**.**67**
*M. laxa*	2B1-A5	71	406.513	42.16	0.96
	2B1-A2-2	74	420.554	42.01	1.00
	MM-B2-A2	78	1.077.540	42.02	2.56
	MM-B4-A3	60	574.503	42.11	1.36
	MM-B4-A4	88	1.146.113	42.00	2.72
	MT-B1-A3-1	76	1.097.739	42.01	2.61
	NI-B3-A2	75	1.098.551	42.06	2.61
	TB1-A4-2	84	1.127.632	41.99	2.68
	**Average**	**75.75**	**747**.**419**	**42**.**04**	**2**.**06**

### Genome Synteny within and between *M. fructicola* and *M. laxa* Species

**Fig. 4.**
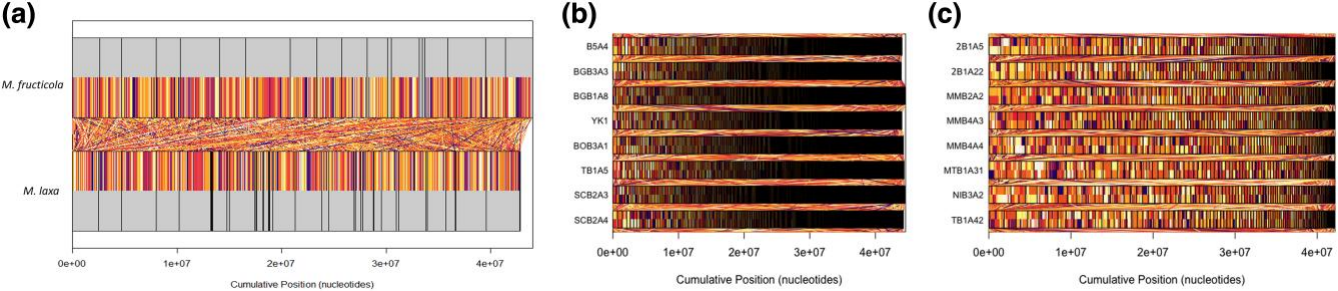
Synteny within and between *M. fructicola* and *M. laxa* species. Alignment of (*a*) reference-based of Ti-B3-A3-2 (*M. fructicola*) and Yildirim-2 (*M. laxa*) and alignment of (*b*) de novo assembled *M. fructicola* and (*c*) de novo assembled *M. laxa* genome assemblies that shows the synteny within and between the species. The analysis identified multiple syntenic hits between the genomes, represented in the figure as linear connections colored uniquely for each hit. Notably, the color-coded linear connections denote regions of the genome where homologous sequences are present in both species, indicating conservation of genomic structure and evolutionary history.

For a high-resolution genomic comparison, the synteny analyses between the species by using our reference genomes of *M. fructicola* and *M. laxa* as well as within the species by using de novo genome assemblies were obtained. Even though the reference genome sizes were similar for these two closely related species (44 Mb for *M. fructicola* and 42 Mb for *M. laxa*), the genomes of Ti-B3-A3–2 (*M. fructicola*) and Yildirim-1 (*M. laxa*) were highly dissimilar with only 41% hits and 7,310 synteny blocks between the genomes (fig. 4*a*). On the other hand, the synteny within the species showed substantial identity between the genomes with ∼50,000 (for *M. fructicola*) and 30,000 (for *M. laxa*) thousands of synteny blocks between the de novo assembled genomes (fig. 4*b* and *c*). Pairwise comparison of synteny statistics including the sequence blocks, percent hits, and the number of synteny blocks between the de novo assembled genomes is presented in supplementary tables S5 and S6, Supplementary Material online, for both species.

## Discussion

Characterization of the structural variations in each species is a crucial step in understanding the adaptive evolutionary processes (Lowry and Willis 2010), but these variants remain unexplored for many phytopathogenic fungi. In many other organisms, it has now been realized that these variants are not only abundant in the genomes, but are also responsible for many phenotypic changes (Wang, et al. 2020; Zhao and Gibbons 2018). To date, the definition, as well as detection of these variants, has been a challenge. Originally, these variants were defined as INDELs and inversions with sizes greater than 1 kb (Feuk , Marshall, et al. 2006). Later, with the advancement in sequencing technologies, and with researchers routinely sequencing many genomes of model and nonmodel organisms, the definition of SVs has also evolved. Currently, the definition of the SVs has been elaborated as variants that change the size, copy number, location, orientation, and sequence content with a size as small as >50 bp (Escaramís, et al. 2015). The challenge now has been turned into the discovery of the extent of SVs and their role in phenotypic changes and evolutionary processes along the tree of life. The number of tools to detect SVs has been increasing (Lei, et al. 2022); however, the precise detection of SVs is highly challenging based on the diverse forms of these variants. Thus, combinational approaches that comprise multiple tools need to be preferred to characterize the genome-wide SVs for a given genomic data set.

In this study, a total of 266.618 and 190.599 SNPs and 1,540 and 918 SV based on variant calling via combinational bioinformatics approaches have been detected for two closely related *M. fructicola* and *M. laxa* species, respectively. In general, the number of both SNPs and SVs was ∼1.5 times higher in *M. fructicola* genomic data set. During our field trips performed in the summer of 2018 for brown rot agents in peach, it was observed that *M. fructicola* species was dominant in our collection and the presence of two identified species (*M. fructicola* and *M. laxa*) varied in different regions of Turkey (e.g., *M. laxa* species were not found in the Black Sea region) (Ozkilinc et al. 2020). It is possible that isolates of *M. fructicola* species have been exposed to possible different selective pressures from both climatic and agricultural practices in different geographical regions. The presence of more variants in *M. fructicola* genome structures than *M. laxa* may be related to the prevalence of the species and the effect of possible selection factors. On the other hand, technically, a higher number of variants in *M. fructicola* isolates might also be due to quality differences of the reference genomes used (e.g., higher number of scaffolds with small sizes in *M. laxa* reference genome making large SV discovery more difficult). However, given the strict filtering applied and the use of multiple tools for variant discovery, we would anticipate that the disparity in the number of variants detected between the two species would be minimally impacted by the quality of their reference genomes. The extent and distribution of SNPs and SVs were highly conserved within the species and presented high diversity between the species. In addition to the variant content, an almost 2.5 times higher Ts/Tv ratio in *M. laxa* in comparison with *M. fructicola* might be an indication of potential divergent evolutionary processes for these closely related species even if the number of isolates studied is low. Moreover, only 41% of similarity hit between *M. fructicola* and *M. laxa* reference genomes further confirmed the potential divergence between these closely related species. For SVs, INDELs were the most commonly occurring type for both species and the number of duplication and inversion events was extremely low for both species (table 1). Interestingly, some of the scaffolds (e.g., scaffold 20 for *M. fructicola* and scaffolds 30, 35, and 38 for *M. laxa*) were highly conserved within species, and only one variant was observed only in one isolate per species. Plant fungal pathogens may harbor the so-called accessory chromosomes which carry genes that are not essential to survival (e.g., pathogenicity and virulence-related genes), and these chromosomes may show presence/absence variation (PAV) within the individuals of the same species (Witte, et al. 2021). Thus, the unique variants coming from a single isolate raise the question of whether other isolates carry the corresponding scaffold/region or not. In other words, do these regions show PAV for different isolates? However, the synteny analyses (fig. 4*b* and *c*) and total genome sizes (supplementary table S1, Supplementary Material online) have indicated that the genomes are highly similar in structure and content among the isolates within each species. Nevertheless, the knowledge of the chromosome number for *Monilinia* species does not exist and considering the variation discovered within this study and the diversity of fungal pathogens present, more studies with a higher number of isolates around the world are required to unravel the detailed genomic properties of these species.

Interestingly, no SVs were present from a large proportion of scaffold 16 (with a size of 1.04 Mb) as well as in many other scaffolds (e.g., scaffolds from 37 to 49 with sizes ranging from 25 to 5 kb) for *M. laxa* isolates. On the other hand, even though some scaffolds harbored a low number of SVs, they were present along with the genomic data set of *M. fructicola* species. The extensive genomic studies have provided many insights into genome-wide adaptive evolutionary processes, and many new hypotheses have been derived. One of the hypotheses states that the repeat-rich/gene-sparse regions in the genome evolve faster in contrast to repeat-poor/gene-rich regions with a so-called two-speed genome model to describe the evolution of plant pathogenic fungi (Dong, et al. 2015). Moreover, this model also explains that the genome structure of the fungal genomes (e.g., localizations of the repeats) might directly affect the distribution of the standing genetic variation based on the complex structures presented by the repetitive elements (Dong, et al. 2015). This, in return, can change the overall genome-wide variation content as a result of different evolutionary mechanisms (e.g., selective sweep). Along these lines, *M. laxa* genome might potentially suit for this “two-speed genome model” considering the highly variable variant rate at different compartments of the genomes. However, our hypothesis is solely based on the variable distribution of the variants and genome-wide localizations of genes and repetitive elements should be further investigated in detail to unravel the potential genome-wide evolutionary mechanisms acting on both species. In addition, genome structural variations may have an impact on responses to different selection pressures, and experimental evolution studies are expected to be very informative in this regard. Our ongoing experimental evolutionary studies on these species will be helpful to explain the biological and evolutionary effects of these structural variations we have discovered and the differences in genomic structure between the species.

Furthermore, predictions of functional annotations of the detected variants presented the potential relevance of both SNPs and SVs based on their locations and types for both species. In this context, most of the SNPs were found in intergenic regions. On the other hand, most of the SVs (57.79% and 61.48% of all SVs for *M. fructicola* and *M. laxa*, respectively) were corresponding to transcript sequences that have a high potential functional effect that might take place in adaptive evolutionary processes. These results show the direct potential effects of SVs in comparison with the SNPs and underlie the potential importance of these SVs in any given phenotype considering their abundance and effects along the genome of both species.

The CNVs were retrieved from the total SVs as deleted and duplicated sequences to further investigate the CNV status for each isolate separately. The results indicated that the size of the CNV matters, and even though *M. laxa* isolates consisted of a lower number of CNVs, the total base pairs in length of the CNVs were more than two times larger in comparison with those in the genomes of *M. fructicola* (with averages of 2.06% and 0.67, respectively) (table 4). The number of deletion events was considerably higher in comparison with the duplication events for both species (in total 564 deletions and 7 duplications for *M. fructicola* and 171 deletions and 3 duplications for *M. laxa*). At the evolutionary level, duplication or deletions are common processes that have the potential to change the gene dosage when the events occur with duplication/deletions of genes (Rice and McLysaght 2017). The “gene dosage effect hypothesis” proposes the direct effect of an imbalanced copy number of genes to a given phenotype with high potential in the change of the product of the given gene (Pritchard and Kola 1999). But the duplicability and the fixation of a gene are highly dependent on many factors including the importance, complexity, and number of alternative spliced forms of the gene (Qian and Zhang 2008). It has been shown that the fixation of gene duplication is mainly driven by positive selection based on the effect of the change in gene dosage (Kondrashov and Kondrashov 2006). Thus, the low number of duplication events might be due to nonfavorable selection processes of the duplicated regions for these species. However, it should also be noted that the filtering parameters for the variant calling were strict to only obtain the true positive variants and these low numbers might also be related to the filtering applied.

This study is the first to comprehensively show the extent, distribution, and potential functional consequences of SVs for within and between of two closely related *M. fructicola* and *M. laxa* species. The variant catalog generated in this study opens doors for many further studies to understand the genomic properties and possible evolutionary outcomes for these economically devastating pathogens. Overall, *M. fructicola* isolates were found to be more SV rich in contrast to *M. laxa* isolates. Unlike *M. fructicola* genomic data set, large genomic proportions of *M. laxa* isolates were highly conserved with no SV which suggest potential differing evolutionary processes for these two closely related species.

## Materials and Methods

### Fungal Genomic DNA Extraction, Whole Genome Sequencing, and Quality Filtering of Illumina Reads

A total of 18 genome data (9 for *M. fructicola* and 9 for *M. laxa*) were used in this study. The fungal isolates used in this study were collected from peach orchards in different locations in Turkey during field studies in 2018 (Ozkilinc et al. 2020). The genome data of five *M. fructicola* and seven *M. laxa* were obtained from a previous work performed by our lab group. The genome data of four *M. fructicola* isolates and six *M. laxa* isolates were obtained from a previous study also performed by our group (Yildiz and Ozkilinc 2020, 2021), whereas the genome data of the others were obtained for this study. Information about sampling region and the source of whole genome sequence data of the isolates with SRA accession numbers in NCBI is provided in supplementary table S7, Supplementary Material online.

Total genomic DNA extraction was carried out from mycelium grown in 50 ml of potato dextrose broth in a rotary shaker at 150 rpm at room temperature using the Norgen Plant/Fungi DNA isolation kit (Norgen, Canada), following the manufacturer's protocol. DNA concentrations were quantified by using Qubit v. 3.0 fluorometer (Thermo Fisher Scientific, USA). Sequencing was performed by Macrogen Inc., sequencing service (South Korea), by using the Illumina TruSeq Nano Library construction kit with 350 bp insert size followed by Illumina NovaSeq 6000 platform with 2 × 150 bp paired-end sequencing.

Trimmed Illumina reads (paired-end 2 × 151 bp) belonging to the selected 16 isolates (eight *M. fructicola*; eight *M. laxa*) were used for all downstream analyses. The low-quality read and adapter removal was performed by using Trimmomatic version 0.36 (Bolger, et al. 2014) following the setting of the parameters as “ILLUMINACLIP:TruseqHT.fa:2:30:10 LEADING:10 TRAILING:10; SLIDINGWINDOW:5:20; MINLEN:151.” The quality statistics of the fastq files were checked with FastQC (Andrews, et al. 2010).

### Genome Assemblies

#### Reference-Based Genome Assembly

Reference-based genome assembly and annotation were performed by the “Reference-based Genome Assembly and Annotation Tool (RGAAT)” (Liu, et al. 2018) for Ti-B3-A3-2 (*M. fructicola*) and Yildirim-1 (*M. laxa*) isolates based on the high mapping rate (>90%) these isolates present. Previously assembled genomes for *M. fructicola* (De Miccolis Angelini, et al. 2019) and *M. laxa* (Landi, et al. 2020) under GenBank accessions “GCA_008692225.1” for *M. fructicola* and “GCA_009299455.1” for *M. laxa* were used as reference genomes. RGAAT uses binary alignment map files (BAM) and reference genome in the FASTA format and updates and outputs the reference genome fasta file by performing coordinate conversion based on variant calling results the tool itself performs. The sequence alignment map (SAM) files required for the tool is obtained by mapping the sequence reads of Ti-B3-A3-2 (*M. fructicola*) and Yildirim-1 (*M. laxa*) to previously published reference genomes for *M. laxa* with BWA MEM version 0.7.17 (Li and Durbin 2009). The SAM files were then converted to BAM files by samtools version 1.12 (Li, et al. 2009), and remapping of the reads was performed by using Stampy version 1.0.32 (Lunter and Goodson 2011) with –bamkeepgoodreads flag.

#### *De Novo* Genome Assemblies

De novo genome assemblies for all isolates were generated by SPADES version 3.11.1 (Bankevich, et al. 2012) with –careful parameter and k-mer range of “21,33,55,77,99,127.” Following the reference-based and de novo assembly, the quality assessments of the genome assemblies for both species were checked by Quast version 5.0.2 (Gurevich, et al. 2013). Moreover, BUSCO version 4 (Seppey, et al. 2019) was used to assess the completeness of each de novo assembled genomes based on the fungi database.

### Mapping of Short Reads to Reference Genomes

The genomes of “Ti-B3-A3-2” for *M. fructicola* (44.02 Mb in size) and “Yildirim-1” for *M. laxa* (42.80 Mb in size) strains obtained by RGAAT (Liu et al. 2018) were used as our references for all downstream analyses. Illumina reads were aligned to the corresponding species’ reference genome obtained by RGAAT using BWA MEM version 0.7.17 (Li and Durbin 2009) to generate SAM files. All SAM files were converted to binary (BAM) files and sorted by PICARD tools version 2.23.6 (https://broadinstitute.github.io/picard/). PICARD TOOLS version 2.23.6 was used for marking and removing the duplicates from the BAM files. Qualimap version 2.2.1 was then used to generate summary statistics of each alignment file (García-Alcalde, et al. 2012).

### SNP, SV and CNV Calling, Selection of Variants, and Quality Filtering

Following the alignment, the Genome Analysis Toolkit (GATK) version 4.2-0 (McKenna, et al. 2010) was used to call SNPs between Illumina reads and the given references. First, the module “HaplotypeCaller” with the “-ploidy 1” option was used to create genome variant call format (gVCF) files and gVCFs were combined by “CombineGVCFs.” Then, the combined gvcf file is genotyped by the “GenotypeGVCFs” module. The GATK module “SelectVariants” were used to select SNPs only. Then, the module “VariantFiltration” was used to quality filter the SNPs (based on QD < 2.0 || FS > 60.0 || MQ < 40.0 || MQRankSum < −12.5 || ReadPosRankSum < −8.0) individually.

The combination of LUMPY Express version 0.2.13 (Layer et al. 2014) and GATK version 4.2-0 (McKenna, et al. 2010) were used to call structural variations. First, the “SelectVariants” module of GATK was used on the genotyped vcf obtained above to select INDELs with sizes >50 bp. Then, the “VariantFiltration” was used to quality filter the INDELs (>50 bp) with “QD < 2.0 || FS > 200.0 || ReadPosRankSum < −20.0.” Then, the LUMPY Express was used to call large SVs (deletions, duplications, and inversions) for each isolate using the alignment (BAM) files obtained above following the workflow on GitHub (https://github.com/arq5x/lumpy-sv). In summary, discordant paired-end alignments were extracted with samtools view, filtering out reads by flag 1294, and split-read alignments were extracted by extractSplitReads_BwaMem script implemented in LUMPY (Layer, et al. 2014). Both extracted alignment reads were sorted and then provided as input to the lumpyexpress utility along with the original BAM file to obtain SVs. Then, a series of strict filtering has been applied to eliminate the false-positive variants. The “vcffilter” tool of vcflib version 1.0.2 (Garrison et al. 2021) was used to filter out the calls if SVTYPE is not “DEL” “DUP” or “INV” with PE < 3 and “IMPRECISE” with a negative confidence interval (CIPOS95) value. The resulting vcf outputs were merged with the vcf-merge of vcftools version 0.1.17 (Danecek, et al. 2011) for each species individually. Then bcftools isec of the bedtools suite (Quinlan and Hall 2010) was used to find intersects of SVs called by GATK and LUMPY, and one of the variants present in both vcf output was kept for downstream analyses.

In addition, the CNVs are obtained as deletions and duplications with PE > 3 following the additional filtration on “IMPRECISE” calls with a negative confidence interval value (CIPOS95) from each vcf output of lumpyexpress with vcffilter tool of vcflib version 1.0.2 again (Garrison, et al. 2021). The resulting vcf files were merged with the vcf-merge of vcftools version 0.1.17 (Danecek, et al. 2011) for each species and isolates separately.

### The Distribution and Prediction of Functional Effects of Variants Along the Genomes of *M. fructicola* and *M. laxa*

The variant annotation, distribution based on the scaffolds, and functional effect of final VCFs (SNPs and SVs) for each species were predicted by SnpEff version 5.0 (Cingolani, et al. 2012). The reference genomes for both *M. fructicola* and *M. laxa* were not present in the database of SnpEff version 5.0; thus, the reference genomes (under GenBank accessions “GCA_008692225.1” for *M. fructicola* and “GCA_009299455.1” for *M. laxa*) were built manually.

The variant distribution for both species was then investigated in detail after normalizing the data set considering the number of variants based on the corresponding scaffold's length as following: (relative abundance, %) = (100 × number of variants on the scaffold)/the length of the scaffold.

### Comparison of Genomes by Synteny

The DECIPHER package (Wright 2016) under R version 4.1.1 (R Core Team 2013) was used to align genomes used in this study to investigate the synteny between and within *M. fructicola* and *M. laxa* species. First, reference genomes of Ti-B3-A3-2 (*M. fructicola*) and Yildirim-1 (*M. laxa*) were aligned to investigate the synteny between these two closely related species. Then, each de novo assembled genomes for both species separately were aligned to reveal the synteny within the species. For synteny analyses, the genomes were connected to the manually constructed SQLite database via the RSQLite package (James and Falcon 2011), and then, the “FindSynteny” function of DECIPHER package (Wright 2016) was used to identify the synteny between the genomes. Finally, the “neighbor” option of the “plot” function in DECIPHER package (Wright 2016) was used to visualize the “synteny” object for each synteny analysis.

## Supplementary Material

evad085_Supplementary_Data

## Data Availability

All the data generated and used in this study are available in NCBI GenBank under the BioProject with the accession number PRJNA846280. In addition, the whole genome sequencing data for all nine *M. fructicola* isolates used in this study are found in NCBI GenBank under the SRA accession numbers SRS13297834, SRS13297843, SRS13297853, SRS13297830, SRS13297847, SRS13297850, SRS13297832, SRS13297851, and SRS13297852, along with isolate names. The whole genome sequencing data for all nine *M. laxa* isolates used in this study are found in NCBI GenBank under the SRA accession numbers SRS13297846, SRS13297836, SRS13297837, SRS13297838, SRS13297840, SRS13297841, SRS13297843, SRS13297844, and SRS13297845, along with isolate names.
