# Supplementary material for: Genome-Wide Discovery of Structural Variants Reveals Distinct Variant Dynamics for Two Closely Related Monilinia Species
Source: Genome Biol Evol. 2023 May 22;15(6):evad085. doi: 10.1093/gbe/evad085 (PMC10234401; doi:10.1093/gbe/evad085)
Supplement: evad085_Supplementary_Data [file evad085_supplementary_data.zip › Supplementary Tables_edited_final.docx]

**Table S1.** Basic assembly statistics and averages of the number of contigs, N50, L50, N’s per 100 kb, GC content (%), length, and BUSCO complete gene percentages based on fungi database for the *de novo* assembled genomes of each isolate.

| Species | Isolate Code | Number of Contigs | N50 (bp) | L50 | N’s per 100 kb | GC Content (%) | Total Length (Mb) | BUSCO Complete Gene Percentage (%) |
| --- | --- | --- | --- | --- | --- | --- | --- | --- |
| *Monilinia fructicola* | BG-B3-A1 | 691 | 120683 | 108 | 9.38 | 40.97 | 43.84 | 99.30 |
|  | SC-B2-A3 | 828 | 111215 | 120 | 8.87 | 40.81 | 44.18 | 99.30 |
|  | SC-B2-A4 | 586 | 146461 | 88 | 8.43 | 40.81 | 44.20 | 99.30 |
|  | B5-A4 | 595 | 154409 | 88 | 8.48 | 40.95 | 43.91 | 99.30 |
|  | T-B1-A5 | 610 | 140742 | 92 | 9.12 | 40.77 | 44.23 | 99.20 |
|  | YK-1 | 666 | 129314 | 100 | 8.83 | 40.96 | 43.88 | 99.20 |
|  | BG-B1-A8 | 760 | 121744 | 119 | 7.37 | 40.63 | 44.51 | 99.20 |
|  | BO-B3-A1 | 701 | 130119 | 104 | 8.42 | 40.81 | 44.18 | 99.30 |
|  | **Average** | **679** | **131835** | **102** | **8.61** | **40.83** | **44.11** | **99.26** |
| *Monilinia laxa* | 2B1-A2-2 | 226 | 412408 | 32 | 2.92 | 40.20 | 42.01 | 98.90 |
|  | Ni-B3-A2 | 181 | 653047 | 20 | 3.50 | 40.18 | 42.06 | 98.80 |
|  | MM-B4-A4 | 117 | 631930 | 22 | 4.07 | 40.19 | 42.00 | 99.10 |
|  | MT-B1-A3-1 | 103 | 673182 | 20 | 3.08 | 40.20 | 42.01 | 99.10 |
|  | T-B1-A4-2 | 124 | 633580 | 23 | 3.33 | 40.21 | 41.99 | 98.90 |
|  | MM-B2-A2 | 161 | 539125 | 27 | 4.83 | 40.19 | 42.02 | 99.10 |
|  | 2B1-A5 | 163 | 465265 | 28 | 3.53 | 40.10 | 42.16 | 98.90 |
|  | MM-B4-A3 | 168 | 411621 | 32 | 3.21 | 40.14 | 42.11 | 99.10 |
|  | **Average** | **155** | **552519** | **25** | **3.55** | **40.17** | **42.04** | **98.98** |

**Table S2.** Number of reads (in millions), overall properly paired read percentages, and mean coverages with average values based on bam statistics for each isolate based on *M. fructicola* (Ti-B3-A3-2 as reference) and *M. laxa* (Yildirim-1 as reference).

| Isolate Code | Species | Number of Reads (million) | Overall Mapped Reads  Percentage (%) | Mean Coverage |
| --- | --- | --- | --- | --- |
| B5-A4 | *M. fructicola* | 32.22 | 87.14 | 95.09 |
| BG-B3-A1 | *M. fructicola* | 38.43 | 84.77 | 110.70 |
| BG-B1-A8 | *M. fructicola* | 26.61 | 85.00 | 76.54 |
| YK-1 | *M. fructicola* | 54.98 | 90.07 | 169.46 |
| BO-B3-A1 | *M. fructicola* | 35.15 | 83.70 | 100.05 |
| T-B1-A5 | *M. fructicola* | 54.61 | 90.36 | 167.61 |
| SC-B2-A3 | *M. fructicola* | 37.81 | 84.99 | 109.28 |
| SC-B2-A4 | *M. fructicola* | 36.84 | 92.17 | 115.38 |
|  | **Average** | **39.58** | **87.27** | **118.01** |
| 2B1-A2-2 | *M. laxa* | 36.54 | 98.61 | 216.49 |
| 2B1-A5 | *M. laxa* | 48.28 | 98.01 | 165.99 |
| MM-B4-A3 | *M. laxa* | 35.02 | 96.69 | 118.87 |
| T-B1-A4-2 | *M. laxa* | 37.83 | 98.52 | 130.81 |
| MM-B2-A2 | *M. laxa* | 51.01 | 98.81 | 176.94 |
| MM-B4-A4 | *M. laxa* | 50.90 | 99.01 | 176.91 |
| MT-B1-A3-1 | *M. laxa* | 38.28 | 98.28 | 132.14 |
| NI-B3-A2 | *M. laxa* | 33.31 | 98.92 | 115.77 |
|  | **Average** | **41.39** | **98.35** | **154.24** |

**Table S3.** The length of each scaffold, number of variants, and relative abundance of each variant type for *M. fructicola*.

|  |  | Number of Variants | | Relative Abundance of Variants (%)* | |
| --- | --- | --- | --- | --- | --- |
| Scaffold Name | Length (bp) | SNPs | Structural Variants | SNPs | Structural Variants |
| VICG01000001.1 | 4,238,823 | 25,566 | 162 | 0.603139 | 0.003822 |
| VICG01000002.1 | 3,728,272 | 16,705 | 110 | 0.448063 | 0.00295 |
| VICG01000003.1 | 3,609,855 | 17,651 | 98 | 0.488967 | 0.002715 |
| VICG01000004.1 | 3,332,839 | 17,271 | 85 | 0.518207 | 0.00255 |
| VICG01000005.1 | 2,656,653 | 15,268 | 105 | 0.574708 | 0.003952 |
| VICG01000006.1 | 2,602,481 | 21,574 | 129 | 0.828978 | 0.004957 |
| VICG01000007.1 | 2,592,823 | 15,607 | 99 | 0.601931 | 0.003818 |
| VICG01000008.1 | 2,576,762 | 17,134 | 82 | 0.664943 | 0.003182 |
| VICG01000009.1 | 2,530,796 | 15,957 | 103 | 0.630513 | 0.00407 |
| VICG01000010.1 | 2,442,597 | 13,231 | 68 | 0.541678 | 0.002784 |
| VICG01000011.1 | 2,335,009 | 14,766 | 104 | 0.632374 | 0.004454 |
| VICG01000012.1 | 2,285,153 | 10,758 | 58 | 0.470778 | 0.002538 |
| VICG01000013.1 | 2,201,684 | 10,515 | 60 | 0.477589 | 0.002725 |
| VICG01000014.1 | 2,106,460 | 17,026 | 72 | 0.808275 | 0.003418 |
| VICG01000015.1 | 1,968,927 | 13,945 | 93 | 0.708254 | 0.004723 |
| VICG01000016.1 | 1,888,073 | 13,253 | 74 | 0.701933 | 0.003919 |
| VICG01000017.1 | 355,621 | 2,278 | 6 | 0.64057 | 0.001687 |
| VICG01000018.1 | 311,593 | 893 | 5 | 0.286592 | 0.001605 |
| VICG01000019.1 | 255,115 | 7,219 | 27 | 2.829704 | 0.010583 |
| VICG01000020.1 | 28,364 | 1 | 0 | 0.003526 | 0 |

* Relative Abundance = (100* “Number of Variant Type”)/ “Length of the Scaffold”.

**Table S4.** The length of each scaffold, number of variants, and relative abundance of each variant type for *M. laxa*.

|  |  | Number of Variants | | Relative Abundance of Variants (%)* | |
| --- | --- | --- | --- | --- | --- |
| Scaffold Name | Length (bp) | SNPs | Structural Variants | SNPs | Structural Variants |
| VIGI01000001.1 | 3,307,454 | 10,489 | 71 | 0.317132 | 0.002147 |
| VIGI01000002.1 | 3,224,414 | 11,520 | 48 | 0.357274 | 0.001489 |
| VIGI01000003.1 | 3,034,541 | 8,984 | 54 | 0.296058 | 0.00178 |
| VIGI01000004.1 | 2,895,099 | 12,070 | 52 | 0.416911 | 0.001796 |
| VIGI01000005.1 | 2,663,264 | 10,711 | 70 | 0.402176 | 0.002628 |
| VIGI01000006.1 | 2,508,015 | 17,360 | 73 | 0.692181 | 0.002911 |
| VIGI01000007.1 | 2,477,070 | 8,438 | 48 | 0.340644 | 0.001938 |
| VIGI01000008.1 | 2,449,422 | 9,495 | 40 | 0.387642 | 0.001633 |
| VIGI01000009.1 | 2,386,254 | 12,677 | 62 | 0.531251 | 0.002598 |
| VIGI01000010.1 | 2,193,015 | 6,921 | 44 | 0.315593 | 0.002006 |
| VIGI01000011.1 | 2,186,364 | 7,222 | 44 | 0.33032 | 0.002012 |
| VIGI01000012.1 | 2,137,041 | 7,195 | 41 | 0.33668 | 0.001919 |
| VIGI01000013.1 | 1,775,491 | 3,770 | 39 | 0.212336 | 0.002197 |
| VIGI01000014.1 | 1,383,779 | 8,486 | 27 | 0.613248 | 0.001951 |
| VIGI01000015.1 | 1,359,562 | 7,420 | 25 | 0.545764 | 0.001839 |
| VIGI01000016.1 | 1,047,848 | 7,069 | 21 | 0.674621 | 0.002004 |
| VIGI01000017.1 | 933,894 | 4,165 | 19 | 0.445982 | 0.002034 |
| VIGI01000018.1 | 743,087 | 1,850 | 14 | 0.248961 | 0.001884 |
| VIGI01000019.1 | 643,592 | 2,970 | 21 | 0.461472 | 0.003263 |
| VIGI01000020.1 | 601,272 | 3,666 | 27 | 0.609707 | 0.00449 |
| VIGI01000021.1 | 547,662 | 8,686 | 20 | 1.586015 | 0.003652 |
| VIGI01000022.1 | 446,339 | 2,084 | 16 | 0.46691 | 0.003585 |
| Table S4 continued... | | | | | |
| VIGI01000023.1 | 302,711 | 2,216 | 2 | 0.732051 | 0.000661 |
| VIGI01000024.1 | 286,346 | 3,324 | 11 | 1.160833 | 0.003842 |
| VIGI01000025.1 | 233,785 | 2,885 | 14 | 1.23404 | 0.005988 |
| VIGI01000026.1 | 165,609 | 1,118 | 2 | 0.675084 | 0.001208 |
| VIGI01000027.1 | 113,273 | 79 | 2 | 0.069743 | 0.001766 |
| VIGI01000028.1 | 84,137 | 1,966 | 4 | 2.336665 | 0.004754 |
| VIGI01000029.1 | 70,559 | 3 | 0 | 0.004252 | 0 |
| VIGI01000030.1 | 69,030 | 1 | 0 | 0.001449 | 0 |
| VIGI01000031.1 | 62,789 | 569 | 0 | 0.90621 | 0 |
| VIGI01000032.1 | 58,386 | 766 | 0 | 1.311958 | 0 |
| VIGI01000033.1 | 62,389 | 0 | 4 | 0 | 0.006411 |
| VIGI01000034.1 | 51,566 | 4 | 1 | 0.007757 | 0.001939 |
| VIGI01000035.1 | 46,913 | 1 | 0 | 0.002132 | 0 |
| VIGI01000036.1 | 32,905 | 111 | 2 | 0.337335 | 0.006078 |
| VIGI01000037.1 | 25,198 | 496 | 0 | 1.96841 | 0 |
| VIGI01000038.1 | 24,684 | 1 | 0 | 0.004051 | 0 |
| VIGI01000039.1 | 23,191 | 121 | 0 | 0.521754 | 0 |
| VIGI01000040.1 | 23,152 | 491 | 0 | 2.120767 | 0 |
| VIGI01000041.1 | 20,823 | 143 | 0 | 0.686741 | 0 |
| VIGI01000042.1 | 20,791 | 554 | 0 | 2.664614 | 0 |
| VIGI01000043.1 | 17,556 | 105 | 0 | 0.598086 | 0 |
| VIGI01000044.1 | 16,968 | 295 | 0 | 1.738567 | 0 |
| VIGI01000045.1 | 16,452 | 700 | 0 | 4.254802 | 0 |
| VIGI01000046.1 | 14,865 | 162 | 0 | 1.089808 | 0 |
| VIGI01000047.1 | 12,908 | 221 | 0 | 1.712117 | 0 |
| VIGI01000048.1 | 8,084 | 510 | 0 | 6.308758 | 0 |
| VIGI01000049.1 | 5,295 | 509 | 0 | 9.612842 | 0 |

**Table S5.** Pairwise comparison of synteny statistics including the sequence blocks (seqs), percent hits (% hits), and the number of synteny blocks (blocks) between the de novo assembled genomes of *M. fructicola* isolates.

| Isolate Code | B5-A4 | BG-B3-A3 | BG-B1-A8 | YK-1 | BO-B3-A1 | T-B1-A5 | SC-B2-A3 | SC-B2-A4 |
| --- | --- | --- | --- | --- | --- | --- | --- | --- |
| B5-A4 | 906 seqs | 119% hits | 120% hits | 119% hits | 119% hits | 119% hits | 119% hits | 119% hits |
| BG-B3-A3 | 50771 blocks | 987 seqs | 120% hits | 120% hits | 120% hits | 119% hits | 120% hits | 120% hits |
| BG-B1-A8 | 50004 blocks | 49904 blocks | 1230 seqs | 121% hits | 120% hits | 120% hits | 120% hits | 120% hits |
| YK-1 | 50825 blocks | 50081 blocks | 53871 blocks | 1053 seqs | 119% hits | 119% hits | 119% hits | 119% hits |
| BO-B3-A1 | 50466 blocks | 49985 blocks | 53558 blocks | 49424 blocks | 1057 seqs | 120% hits | 121% hits | 120% hits |
| T-B1-A5 | 50071 blocks | 49778 blocks | 53818 blocks | 49132 blocks | 51300 blocks | 988 seqs | 120% hits | 120% hits |
| SC-B2-A3 | 51513 blocks | 51259 blocks | 55326 blocks | 50992 blocks | 52173 blocks | 52413 blocks | 1673 seqs | 120% hits |
| SC-B2-A4 | 50609 blocks | 49732 blocks | 53772 blocks | 49116 blocks | 50962 blocks | 51275 blocks | 51769 blocks | 942 seqs |

**Table S6.** Pairwise comparison of synteny statistics including the sequence blocks (seqs), percent hits (% hits), and the number of synteny blocks (blocks) between the de novo assembled genomes of *M. laxa* isolates.

| Isolate Code | 2B1-A5 | 2B1-A2-2 | MM-B2-A2 | MM-B4-A3 | MM-B4-A4 | MT-B1-A3-1 | NI-B3-A2 | T-B1-A4-2 |
| --- | --- | --- | --- | --- | --- | --- | --- | --- |
| 2B1-A5 | 267 seqs | 122% hits | 121% hits | 122% hits | 122% hits | 121% hits | 121% hits | 122% hits |
| 2B1-A2-2 | 29647 blocks | 558 seqs | 120% hits | 121% hits | 120% hits | 120% hits | 120% hits | 121% hits |
| MM-B2-A2 | 28621 blocks | 28111 blocks | 297 seqs | 120% hits | 120% hits | 120% hits | 119% hits | 120% hits |
| MM-B4-A3 | 29249 blocks | 28700 blocks | 27587 blocks | 286 seqs | 120% hits | 120% hits | 120% hits | 121% hits |
| MM-B4-A4 | 28812 blocks | 28203 blocks | 27450 blocks | 28229 blocks | 200 seqs | 119% hits | 119% hits | 120% hits |
| MT-B1-A3-1 | 28799 blocks | 27973 blocks | 27465 blocks | 28006 blocks | 27105 blocks | 174 seqs | 119% hits | 120% hits |
| NI-B3-A2 | 28604 blocks | 28106 blocks | 27068 blocks | 27961 blocks | 27172 blocks | 27133 blocks | 515 seqs | 120% hits |
| T-B1-A4-2 | 29084 blocks | 28605 blocks | 27913 blocks | 28476 blocks | 27845 blocks | 27476 blocks | 27640 blocks | 291 seqs |

**Table S7.** Fungal isolates used in this study, their hosts, sampling sites in Turkey, next generation sequencing (NGS) data sources, and their SRA accession numbers in NCBI.

| Species | Isolate Code | Host | City/Geographic Region | NGS Resource | SRA Accession Number |
| --- | --- | --- | --- | --- | --- |
|  | Ti-B3-A3-2 | Peach | Izmir/Aegean | Yildiz and Ozkilinc, 2021 | SRS13297834 |
| *Monilinia fructicola* | BG-B3-A1 | Peach | Bursa/Marmara | This study | SRS13297853 |
|  | SC-B2-A3 | Peach | Samsun/Black Sea | This study | SRS13297851 |
|  | SC-B2-A4 | Peach | Samsun/Black Sea | Yildiz and Ozkilinc, 2021 | SRS13297852 |
|  | B5-A4 | Peach | Canakkale/Marmara | Yildiz and Ozkilinc, 2021 | SRS13297831 |
|  | T-B1-A5 | Peach | Izmir/Aegean | Yildiz and Ozkilinc, 2021 | SRS13297832 |
|  | YK-1 | Peach | Izmir/Aegean | Yildiz and Ozkilinc, 2021 | SRS13297847 |
|  | BG-B1-A8 | Peach | Bursa/Marmara | This study | SRS13297830 |
|  | BO-B3-A1 | Peach | Bursa/Marmara | This study | SRS13297850 |
|  | Yildirim-1 | Peach | Bursa/Marmara | Yildiz and Ozkilinc, 2021 | SRS13297846 |
| *Monilinia laxa* | 2B1-A2-2 | Peach | Canakkale/Marmara | This study | SRS13297836 |
|  | Ni-B3-A2 | Peach | Nigde/Central | Yildiz and Ozkilinc, 2020 | SRS13297845 |
|  | MM-B4-A4 | Peach | Mersin/Mediterranean | Yildiz and Ozkilinc, 2021 | SRS13297843 |
|  | MT-B1-A3-1 | Peach | Mersin/Mediterranean | Yildiz and Ozkilinc, 2021 | SRS13297844 |
|  | T-B1-A4-2 | Peach | Izmir/Aegean | Yildiz and Ozkilinc, 2021 | SRS13297840 |
|  | MM-B2-A2 | Peach | Mersin/Mediterranean | Yildiz and Ozkilinc, 2021 | SRS13297841 |
|  | 2B1-A5 | Peach | Canakkale/Marmara | Yildiz and Ozkilinc, 2021 | SRS13297837 |
|  | MM-B4-A3 | Peach | Mersin/Mediterranean | This study | SRS13297838 |
