# Supplementary material for: Genome-Wide Discovery of Structural Variants Reveals Distinct Variant Dynamics for Two Closely Related Monilinia Species
Source: Genome Biol Evol. 2023 May 22;15(6):evad085. doi: 10.1093/gbe/evad085 (PMC10234401; doi:10.1093/gbe/evad085)
Supplement: evad085_Supplementary_Data [file evad085_supplementary_data.zip › Supplementary Data Legends.docx]

**Supplementary Data**

**Table S1.** Basic assembly statistics and averages of the number of contigs, N50, L50, N’s per 100 kb, GC content (%), length, and BUSCO complete gene percentages based on fungi database for the *de novo* assembled genomes of each isolate.

**Table S2.** Number of reads (in millions), overall properly paired read percentages, and mean coverages with average values based on bam statistics for each isolate based on *M. fructicola* (Ti-B3-A3-2 as reference) and *M. laxa* (Yildirim-1 as reference).

**Table S3.** The length of each scaffold, number of variants, and relative abundance of each variant type for *M. fructicola*.

**Table S4.** The length of each scaffold, number of variants, and relative abundance of each variant type for *M. laxa*.

**Table S5.** Pairwise comparison of synteny statistics including the sequence blocks (seqs), percent hits (% hits), and the number of synteny blocks (blocks) between the *de novo* assembled genomes of *M. fructicola* isolates.

**Table S6.** Pairwise comparison of synteny statistics including the sequence blocks (seqs), percent hits (% hits), and the number of synteny blocks (blocks) between the *de novo* assembled genomes of *M. laxa* isolates.

**Table S7.** Fungal isolates used in this study, their hosts, sampling sites in Turkey, next generation sequencing (NGS) data sources and their SRA accession numbers in NCBI.

**Fig. S1.** Distribution of total contig lengths (y-axis) at different read coverage depths (x-axis, grouped in bins) for *de novo* assembled a) *M. fructicola* and, b) *M. laxa* genomes.
