# Supplementary figures and images for: Genome-Wide Discovery of Structural Variants Reveals Distinct Variant Dynamics for Two Closely Related Monilinia Species
Source: Genome Biol Evol. 2023 May 22;15(6):evad085. doi: 10.1093/gbe/evad085 (PMC10234401; doi:10.1093/gbe/evad085)

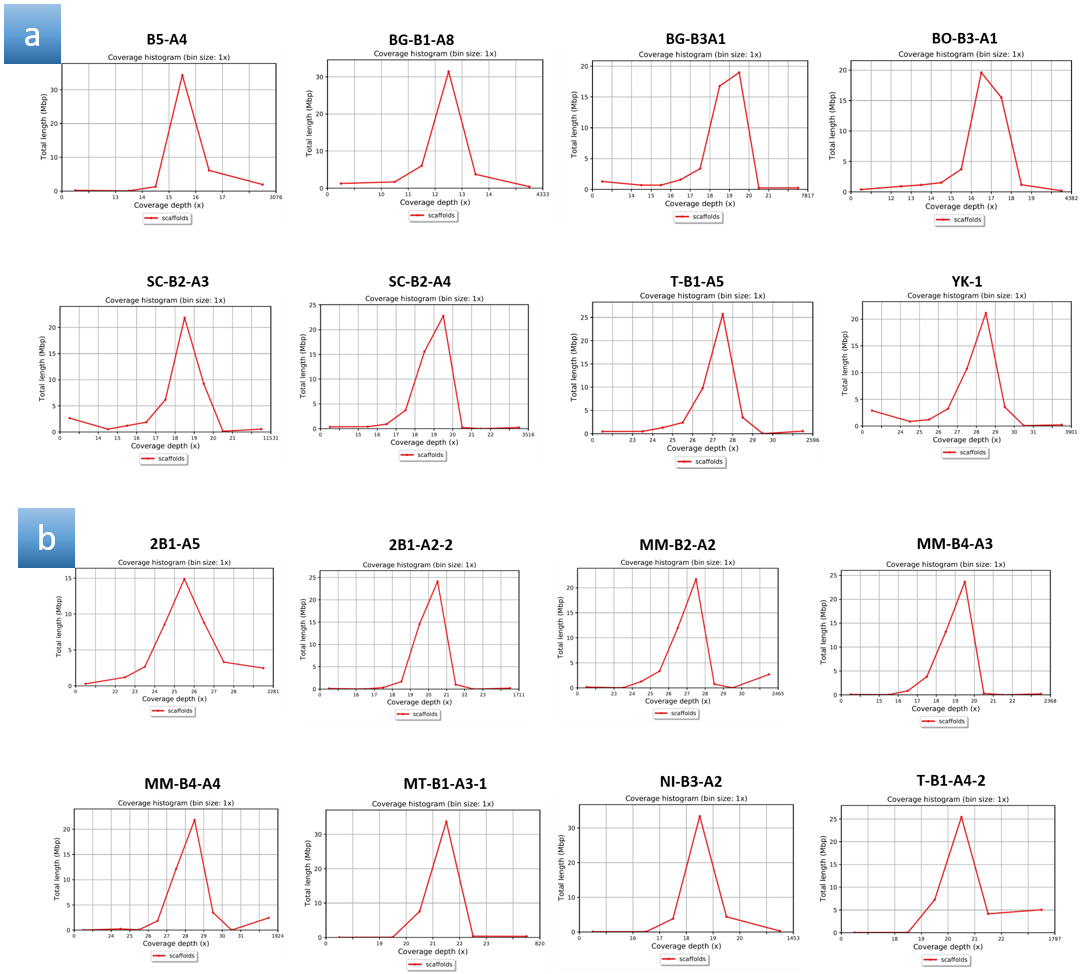

Supplement: evad085_Supplementary_Data [file evad085_supplementary_data.zip › Figure S1.tif]
